# How Much Does a Home Care Nursing Visit Cost? A National Micro-Costing Study from the AIDOMUS-IT Project

**DOI:** 10.3390/nursrep16060180

**Published:** 2026-05-26

**Authors:** Marco Di Nitto, Paolo Landa, Paolo Iovino, Rosaria Alvaro, Alessandra Burgio, Valeria Caponnetto, Stefano Domenico Cicala, Giancarlo Cicolini, Manuele Cesare, Loreto Lancia, Duilio Fiorenzo Manara, Ilaria Marcomini, Beatrice Mazzoleni, Alvisa Palese, Laura Rasero, Gennaro Rocco, Francesco Zaghini, Loredana Sasso, Annamaria Bagnasco

**Affiliations:** 1 Department of Health Sciences, University of Genoa, 16132 Genoa, Italy; marco.dinitto@unige.it (M.D.N.); l.sasso@unige.it (L.S.); annamaria.bagnasco@unige.it (A.B.); 2 Département des Opérations et Systèmes de Décision, Faculté des Sciences de L’Administration, Université Laval, Quebec, QC G1V 0A6, Canada; paolo.landa@fsa.ulaval.ca; 3 Axe Santé des Populations et Pratiques Optimales en Santé, Centre de Recherche du Centre Hospitalier Universitaire de Québec, Quebec, QC G1V 2M2, Canada; 4 Centre Interuniversitaire de Recherche sur les Réseaux d’Entreprise, la Logistique et le Transport (CIRRELT), Quebec, QC G1K 7P4, Canada; 5 Department of Health Sciences, University of Florence, 50143 Florence, Italy; l.rasero@unifi.it; 6 Scientific Committee CERSI-FNOPI (Centro di Eccellenza per la Ricerca e lo Sviluppo dell’Infermieristica—Federazione Nazionale degli Ordini delle Professioni Infermieristiche), 00184 Rome, Italy; rosaria.alvaro@uniroma2.it (R.A.); manara.duilio@unisr.it (D.F.M.); 7 Department of Biomedicine and Prevention, Faculty of Medicine, University of Rome Tor Vergata, 00133 Rome, Italy; francesco.zaghini@uniroma2.eu; 8 National Institute of Statistics—ISTAT, 00184 Rome, Italy; 9 Department of Clinical Medicine, Public Health, Life and Environmental Sciences, University of L’Aquila, 67100 L’Aquila, Italy; valeria.caponnetto@univaq.it (V.C.); loreto.lancia@univaq.it (L.L.); 10 Statistics and Healthcare Information Flows, National Agency for Regional Health Services—AGENAS, 00187 Rome, Italy; cicala@agenas.it; 11 Department of Innovative Technologies in Medicine & Dentistry, University of Chieti-Pescara “G. d’Annunzio”, 66013 Chieti, Italy; g.cicolini@unich.it; 12 Gemelli IRCCS University Hospital Foundation, Largo Agostino Gemelli 8, 00168 Rome, Italy; manuele.cesare@policlinicogemelli.it; 13 Section of Hygiene, Department of Life Sciences and Public Health, Catholic University of the Sacred Heart, 00168 Rome, Italy; 14 Center for Nursing Research and Innovation, Faculty of Medicine and Surgery, Vita-Salute San Raffaele University, 20132 Milan, Italy; marcomini.ilaria@unisr.it; 15 FNOPI (Federazione Nazionale degli Ordini delle Professioni Infermieristiche), 00184 Rome, Italy; beatrice.mazzoleni@hunimed.eu; 16 Department of Biomedical Sciences, Humanitas University, Pieve Emanuele, 20090 Milan, Italy; 17 Rete Interuniversitaria di Ricerca Infermieristica Clinica per l’Innovazione Organizzativa e Formativa (RICI), 50134 Florence, Italy; alvisa.palese@uniud.it; 18 Department of Medicine, University of Udine, 33100 Udine, Italy; 19 Centre of Excellence for Nursing Scholarship c/o Order of the Nursing Professions (OPI) of Rome, 00146 Rome, Italy; genna.rocco@gmail.com; 20 CERSI-FNOPI (Centro di Eccellenza per la Ricerca e lo Sviluppo dell’Infermieristica—Federazione Nazionale degli Ordini delle Professioni Infermieristiche), 00184 Rome, Italy

**Keywords:** home care nursing, micro-costing, health economics, community health services, chronic disease, Italian healthcare system

## Abstract

**Background/Objectives**. Country-level evidence on the economic footprint of home care nursing is still scarce, particularly in systems where tariffs for community-based nursing are lacking. In Italy, recent laws have expanded home care; yet planning and funding remain constrained by the absence of robust micro-costing evidence. **Objectives**. To estimate the accounting cost of home care nursing visits in Italy using a bottom-up micro-costing approach and to identify the main cost drivers influencing expenditure. **Methods**. A multicentre, cross-sectional study was conducted. Data were collected in two phases: (1) a national survey of 3949 home care nurses from 70 Local Health Authorities (April–October 2023), describing workload, travel time, and the most frequently performed activities; and (2) a time-and-motion study of 527 consecutive home visits performed by 83 nurses in three Local Health Authorities (March 2024). Direct costs were estimated from the Italian National Health Service perspective and included nursing time, travel time and transportation, back-office activities, and materials. Personnel costs were derived from national collective labour agreements and inflation-adjusted. A base-case scenario estimated accounting costs directly measured in the study. An extended, illustrative scenario explored the economic value of nursing activities by applying existing outpatient tariffs. Deterministic and probabilistic sensitivity analyses (10,000-iteration Monte Carlo simulation) were performed. **Results**. The mean accounting cost of home care nursing was €27.78 per patient per day. At the provider level, the corresponding daily cost per nurse was €190.00, assuming a mean caseload of 6.84 patients per nurse per shift. In the extended scenario, the imputed economic value of nursing activities increased the estimated daily cost to €120.81 per patient and €826.32 per nurse. Sensitivity analyses identified organizational factors (particularly the number of patients per shift and the number of activities per visit) as the dominant cost drivers, while material and transportation costs had a comparatively limited impact. **Conclusions**. Home care nursing in Italy appears to be delivered at a relatively low accounting cost, with organizational factors playing a greater role than unit prices in determining expenditure. The absence of a dedicated reimbursement framework for nursing activities may result in a substantial under-recognition of the economic value of home-based nursing care. These findings provide preliminary evidence to support workforce planning, reimbursement policies, and the sustainable development of territorial care services.

## 1. Introduction

The demographic and epidemiological transition is profoundly reshaping healthcare demand across high-income countries. Globally, the proportion of older adults is rapidly increasing: by 2030, one in six people worldwide will be aged 60 years or older, and this figure is projected to reach 2.1 billion by mid-century [1]. Italy is at the forefront of this demographic shift, with older adults already accounting for approximately 24% of the population and expected to exceed 35% by 2050 [2]. In parallel, the growing prevalence of chronic diseases accounts for over 80% of national health expenditure, placing increasing pressure on the long-term sustainability of the Italian healthcare system [3]. In response to these trends, Italian health policy has progressively strengthened primary and community-based services as a strategic lever to improve continuity of care and contain avoidable hospitalizations. This process culminated in the 2022 national reform defining new organizational models and standards for territorial care [4]. Consistently, Italian and international evidence shows that home care services are mainly used by very old, frail individuals, often affected by multimorbidity, functional dependence, cognitive impairment, polypharmacy, and complex long-term care needs [5,6,7]. Within this framework, home care nursing (HCN) represents a cornerstone of community-based care delivery, particularly for older adults and people living with chronic or complex conditions.

A growing body of evidence suggests that HCN is associated with improved patient-level outcomes, including enhanced patient experience, greater alignment with person-centred care principles, and reduced hospital readmissions [8,9,10,11]. Beyond patient outcomes, HCN also supports family members and informal caregivers by facilitating shared decision-making and promoting care consistent with patient and family preferences [12].

In recent years, international research has increasingly explored the economic implications of home care and community-based services. Several studies suggest that home-based care may represent a cost-effective or potentially cost-saving model in specific clinical contexts, particularly by reducing hospital utilization and supporting care continuity [13,14,15]. However, the available economic evidence remains heterogeneous and strongly context-dependent, reflecting differences in healthcare system organization, workforce structures, and funding mechanisms [16]. In addition, much of the existing literature relies on modelling approaches, disease-specific analyses, or aggregate costing frameworks, rather than empirically derived estimates based on detailed observation of care processes [17,18].

Despite its recognized clinical and organizational value, the economic footprint of HCN remains poorly defined [14,15]. For example, in Italy, unlike medical home visits, nursing activities delivered at home are not covered by a dedicated national tariff schedule. Home care nursing services are primarily financed by the National Health Service (Servizio Sanitario Nazionale, SSN) and are generally provided free of charge to eligible patients. However, in the absence of a dedicated national tariff for home-based nursing activities, substantial regional heterogeneity exists in the organization, funding, and valuation of home nursing services, limiting the ability of policymakers to plan sustainable workforce expansion and equitable service delivery [4].

Robust, bottom-up cost estimates of HCN are therefore essential to support evidence-informed policy decisions. Micro-costing studies allow detailed identification of resource use and cost drivers and are particularly relevant in settings characterized by high organizational variability. Nevertheless, international evidence remains limited. To date, the most detailed micro-costing analyses of home nursing care have been conducted in South Korea using administrative and activity-based data [19,20], while no comparable national studies have been published for the Italian context. This lack of empirically grounded economic evidence represents a critical gap, particularly considering recent investments in territorial care and the ongoing reorganization of community-based services.

### Aims of the Study

The Assistenza Infermieristica Domiciliare in Italia (AIDOMUS-IT) project was designed to address this gap by providing nationwide, empirically grounded data on home care nursing practice [21]. Building on national survey data and detailed time-and-motion observations, the present secondary economic analysis aims to: (i) estimate the accounting cost of HCN and (ii) identify the main cost drivers influencing expenditure through deterministic and probabilistic sensitivity analyses. As a secondary aim, the study aimed to contextualize the estimated costs of HCN within the broader Italian healthcare system through an illustrative comparison with hospital-based care using publicly available national data.

## 2. Materials and Methods

### 2.1. Study Design

AIDOMUS-IT is a nationwide, observational project aimed at estimating the actual costs of HCN in Italy. The present manuscript is based on two data collections performed within the AIDOMUS-IT project. A bottom-up micro-costing approach was adopted to identify and value the resources consumed during home nursing activities, in line with established costing methodologies in healthcare [18]. The study was articulated in two phases; in Phase 1, the AIDOMUS-IT study involved all home care services in Italy, across 18 of the 21 Italian regions and 70 participating Local Health Authorities (LHA). This phase consisted of a nationwide survey of home care nurses aimed at characterizing the organizational structure of services, workload patterns, travel time, caseload, and the distribution of nursing activities performed in routine practice. In Phase 2, a convenience sample of three LHAs from two regions (Liguria and Tuscany) that had participated in the first phase was selected. The LHAs included in Phase 2 were selected based on feasibility and willingness to participate in the time-and-motion data collection, which required significant local organizational support. Selection was purposive and aimed to capture variability in organizational models of home care delivery. This phase involved a detailed time-and-motion data collection, during which nurses prospectively recorded the duration of visits, travel time and distance, types and number of nursing activities performed, and materials used during a full working shift.

### 2.2. Population and Setting

Eligible participants for Phase 1 included: (i) registered nurses working in home care within the participating LHAs; (ii) patients receiving home care services; and (iii) the primary informal caregiver of the patient, in cases where support was needed to complete the survey. The following participants were excluded: (i) nurses employed in home care services who did not provide direct care (e.g., nurses engaged in front-office or coordination roles); and (ii) patients receiving care from services other than home care (e.g., outpatient clinic services, residential care facilities, or hospital-based programmes) [22,23].

Eligible nurses engaged in direct home care delivery within the participating LHAs had to have at least six months of work experience in the respective home care services and be willing to participate. All home care nurses from the selected LHAs were invited to participate. Nurses who expressed interest in participating were given further instructions on how to complete the questionnaires and were informed about the voluntary nature of participation, data confidentiality, and the absence of any consequences in case of non-participation. Written informed consent was obtained prior to data collection. Although patients and informal caregivers were included in the broader AIDOMUS-IT study to collect complementary information, the present economic analysis relied exclusively on nurse-reported data from Phase 1 and time-and-motion observations from Phase 2.

### 2.3. Data Collection Procedures

Phase 1 was conducted between April and October 2023 using a national survey administered to home care nurses through the LimeSurvey^®^ platform [5,24]. The research team trained local facilitators to support data collection across participating LHAs. These facilitators provided both oral and written information about the study objectives to all invited nurses and guided participants through the survey procedures. The survey collected information on sociodemographic characteristics (e.g., age, gender, education level, postgraduate training, years of experience in home care), workload, organizational features, average duration of home visits, number of visits per shift, travel time, and kilometres travelled in the last shift. In addition, nurses reported the ten most frequently performed home care activities, which were subsequently used to inform the classification framework adopted in Phase 2.

Phase 2 was carried out in March 2024 and focused on the detailed recordings of nursing activities performed during home care visits. The structure and content of the paper-based data collection form were adapted from prior studies [25,26,27]. Nurses completed the form after each visit during a full working shift, recording travel time and distance, type, duration and number of nursing activities, and resources (e.g., materials) used. All completed forms were independently transcribed into an electronic database by two researchers and counterchecked.

### 2.4. Measurement of Nursing Activities

The classification of nursing activities was based on data collected from Phase 1, in which nurses reported the ten most frequently performed home care activities. Given the heterogeneity in terminology, reported activities were mapped to the official list of specialist outpatient activities defined by the Italian Ministry of Health [28]. Activities (n = 10,972) were subsequently grouped into seven categories applying a consensus process involving all members of the team (see authors): (1) managing vascular access, infusion therapy, blood sampling; (2) providing health education; (3) providing advanced wound care; (4) providing simple wound care; (5) monitoring vital signs and measurements; (6) planning, evaluation, and documentation of nursing care; and (7) providing other clinical activities (e.g., catheter and enteral nutrition management). The distribution of the activities reported in Phase 1 was used to derive a weighted average cost of activities, reflecting the relative frequency of each category in routine home care nursing practice. The distribution represents the relative frequency of each nursing activity category across all nursing activities recorded in Phase 1. Specifically, each activity reported in the dataset was classified into one of the predefined service categories. The total number of activities within each category was then divided by the total number of nursing activities overall and expressed as a percentage. Therefore, the distribution reflects how often each type of nursing activity occurred in the dataset, rather than the proportion of home care visits in which the activity was performed.

Weighted activity costs were calculated by combining the relative frequency of each activity category with its corresponding unit cost. For each category *i*, a weighted cost contribution was computed as the product of its relative frequency and unit cost (${Weighted\_cost}_{i}\;=\;{Frequency}_{i}\;\times \;{Unit\_cost}_{i}$). The total cost per home care visit was then obtained by summing the weighted contributions across all activity categories. Unit costs were derived from empirical micro-costing estimates in the base-case scenario and from outpatient tariffs in the extended scenario, used as an illustrative proxy.

Missing data in Phase 1 were limited (151 missing responses out of 3949 observations; 3.82%) and were handled using a complete-case approach without imputation, as the level of missingness was considered unlikely to materially affect the estimated distribution of nursing activities. In Phase 2, missingness primarily concerned material cost data recorded at the visit level. Missing material fields were treated as missing information rather than the absence of material use and were therefore excluded from the estimation of mean material costs per visit.

### 2.5. Costing Approach

The economic analysis was conducted from the perspective of the Italian National Health Service (SSN) and included only direct healthcare costs. Resource use and costs were estimated by integrating data from the two AIDOMUS-IT phases and included personnel time, materials, transportation, and administrative activities associated with home care delivery. Costs were initially estimated at the level of individual home care visits and subsequently aggregated to derive mean daily costs per patient and per nurse, reflecting both service delivery and organizational perspectives. Therefore, patient-level characteristics such as age, clinical complexity, diagnosis, and dependency level were not used to stratify cost estimates. In Phase 2, material costs were estimated at the home visit level rather than at the individual procedure level. This approach was adopted because each home visit could include multiple nursing procedures, and material consumption was recorded as part of the visit documentation. Therefore, missing or incomplete information on materials for specific procedures does not affect the estimation of the average material cost per visit but limits the possibility of allocating material costs precisely to individual procedures. Accordingly, the material cost component should be interpreted as an average visit-level material cost, not as a procedure-specific material cost. The analysis adopts a one-year time horizon (no discounting required, given the short horizon). All costs are expressed in 2024 euros, with personnel wages updated from the 2019–2021 CCNL using the official ISTAT revaluation index and material/transport costs taken from 2024 reference sources (AIFA, ACI). The price date of the analysis is therefore 2024. The complete CHEERS 2022 checklist is provided as Supplementary Material S1. Sample-size considerations are reported in Section 2.2; the Phase 1 sample reflects the convenience enrolment of all home care nurses in the 70 participating LHAs across 18 of the 21 Italian regions, while the Phase 2 sample of 527 visits over 83 nurses in 3 LHAs was determined by feasibility considerations and is consistent with sample sizes adopted in comparable time-and-motion studies.

### 2.6. Personnel Costs

Nursing personnel costs were derived from the 2019–2021 National Collective Labour Agreement for the public healthcare sector (CCNL Sanità, 2019–2021). Based on the mean age distribution (45.65 years) of participating nurses, the band 4 (D4) contractual profile was applied as a representative central-tendency proxy. We acknowledge that this is a simplification: the actual seniority distribution of the participating nurses spans several CCNL bands. To assess the impact of this simplification, a sensitivity analysis was conducted applying alternative bands (D3, D5, and the senior Ds profile) corresponding to plausible cohorts of the participating nurses; results show that the per-minute labour cost varies by approximately ±10% across these alternative bands. The per-minute labour cost was calculated assuming 260 working days per year and 7.2 working hours per day. Personnel costs included time spent delivering care at the patient’s home, travel time, and time allocated to administrative and back-office activities.

### 2.7. Back-Office Activities

Time dedicated to administrative and back-office activities (e.g., documentation, care planning, coordination tasks, and completion of clinical records) was incorporated into the personnel cost estimation. These activities were considered part of the routine nursing workload and were valued using the same per-minute labour cost applied to direct care activities.

### 2.8. Material Costs

Material consumption was recorded during Phase 2 and included more than 1000 distinct items. After data cleaning and reclassification, materials were grouped into six categories: catheters, medical devices, medications, wound care supplies, blood collection materials, and miscellaneous items. Unit prices were obtained from the National Pharmaceutical Formulary of the Italian Medicines Agency (AIFA) and regional public procurement catalogues from Liguria and Tuscany. All material costs were updated to 2024 values, calculated at the visit level and averaged across all observed home visits to derive a mean cost per patient visit.

### 2.9. Transportation Costs

Transportation costs included both the cost of nursing time spent travelling and vehicle-related costs. Distance travelled was valued using the 2024 official cost-per-kilometre rates published by the Automobile Club d’Italia (ACI) [29], which are nationally standardized estimates commonly adopted in public-sector economic evaluations and administrative reimbursement procedures in Italy. Travel time was valued using the nurse’s per-minute labour cost. Based on Phase 1 data, an average cumulative travel time of 34.7 min per shift and a distance of 2.93 km per travel segment were applied. Note that the Phase 2 time-and-motion observations report a different but complementary measure: the median duration of each individual travel segment (10 min, IQR 5–15), which, when multiplied by 7.84 segments per shift, implies a cumulative shift travel time of approximately 78 min, higher than the Phase 1 mean. The residual difference reflects the different measurement constructs (retrospective self-reported total in Phase 1 vs. prospective segment-level observation in Phase 2) and, in part, the convenience sampling of three LHAs in Phase 2 with potentially higher patient density per shift. For consistency with the cost build-up at the daily level, we use the Phase 1 cumulative measure throughout the cost estimation; the Phase 2 segment-level data are used for descriptive characterization only. Daily transportation costs were estimated, assuming an average of 6.84 patients visited per nurse per shift, corresponding to 7.84 travel segments per day (including the return to the workplace).

### 2.10. Costing Scenarios

Because in Italy no official fee schedule exists for nursing activities performed at home, two costing scenarios were considered. The base-case scenario estimated accounting costs using directly measured empirical data, including nursing time, travel time, transportation, back-office activities, and materials. This scenario was intended to reflect the minimum measurable cost of delivering home care nursing from the perspective of the Italian National Health Service.

The extended scenario was developed to provide an illustrative estimate of the economic value of nursing activities currently not covered by a dedicated home care tariff. In this scenario, nursing activities were valued using the maximum tariffs listed in the Italian specialist outpatient fee schedule, which represented the only available standardized national tariff reference. This choice was made to avoid assigning a null value to activities that require professional nursing time, technical competence, and clinical responsibility, while recognizing that these tariffs do not constitute an official reimbursement model for home care nursing.

The base case and the extended scenario do not measure the same construct and therefore are not directly additive. The base case is a bottom-up accounting cost measuring the resources actually consumed in delivering one home visit (nursing time, travel, materials, transport, back office), valued at their respective input prices. The extended scenario, by contrast, is a value-attribution exercise that asks how much the same set of nursing activities would be reimbursed if home-based nursing care were paid through the existing outpatient tariff schedule. Because outpatient tariffs are regulatory prices that bundle clinical labour, ancillary materials, equipment depreciation, and indirect overheads into a single price, they necessarily embed the cost of professional nursing time. Adding the tariff-based valuation to the accounting cost would therefore double-count nursing time. Worked example: a single home visit including three nursing activities (the median observed value) consumes resources valued at €27.78 in the base case. Under a hypothetical national reimbursement model based on the maximum outpatient tariff schedule, the same three activities would be valued at €93.03; the €93.03 is therefore not an additional resource consumed, but rather the regulatory value attributed to the visit if such a tariff schedule existed. The €120.81 figure obtained should be interpreted as the upper-bound regulatory value of the visit under the existing outpatient framework, not as the true production cost of the visit.

In line with Phase 2 observations, the extended scenario assumed an average of three nursing activities per home visit, corresponding to the median number of activities recorded during observed visits. This assumption was therefore empirically grounded in the time-and-motion data. Given the uncertainty surrounding tariff-based valuation and activity intensity, these assumptions were tested through deterministic and probabilistic sensitivity analyses.

The choice of the maximum tariffs as the primary reference reflects the consideration that home care nursing activities frequently involve higher technical complexity than the lowest-tariff comparator, as they often require environmental adaptation, broader patient/caregiver assessment, and the management of comorbidity and context-related challenges that are not present in standardized outpatient settings.

### 2.11. Sensitivity Analysis

Deterministic sensitivity analysis was conducted to assess the impact of key parameters on total costs, varying each input individually while holding others constant. Parameters included the number of patients per shift, the number of activities per visit, nursing labour cost, material cost, and travel time and distance. When empirical variability was unavailable, a ±30% range around the base-case value was applied. Probabilistic sensitivity analysis was performed using a Monte Carlo simulation with 10,000 iterations. Appropriate probability distributions were assigned to model parameters (gamma distributions for cost variables and beta distributions for proportions). Results are reported as mean values and 95% uncertainty intervals.

### 2.12. Ethical Considerations

This study was approved by the Ethics Committee of the Liguria Region (No. 675/2022—DB ID 12844) on 29 November 2022, and, where required, by the local ethics committees of the participating LHA. Participation was voluntary, and all participants were informed about the study objectives and were asked to provide written informed consent prior to completing the questionnaires.

## 3. Results

### 3.1. Sample Characteristics

A total of 3949 home care nurses participated in Phase 1, reporting 10,972 nursing activities (Table 1). The mean age of participants was 46.0 years (SD 10.2), and the majority were female (78.2%). Approximately half of the sample held a bachelor’s degree (46.0%), while 25.9% reported having completed postgraduate training in home or family nursing. The mean length of experience in home care nursing was 8.0 years (SD 8.3).

### 3.2. Characteristics of Home Visits and Nursing Activities

In Phase 2, 527 home visits were recorded, resulting in 44 distinct nursing activities. Characteristics of home visits are presented in Table 2. The median travel time to reach the patient’s home was 10 min (interquartile range [IQR] 5–15), with a median distance of 3.5 km (IQR 2–7). The median number of nursing activities performed per visit was three (IQR 2–4.5). Overall, 59.6% of visits involved three or more activities. The most frequently reported activities included documentation and care planning (88.6%), nursing assessment (51.0%), wound dressing (30.4%), health education (35.1%), and blood sampling (17.5%). These findings reflect the multidimensional nature of home care nursing, combining direct clinical activities with assessment, education, and coordination activities.

### 3.3. Cost of Nursing Activities

Based on the distribution of the 10,972 reported nursing activities, a weighted average cost of activities was derived (Table 3). The estimated mean cost of nursing activities performed during a typical home visit was €31.01. The largest contributions to the weighted cost were clinical care activities (€6.27), vascular access and infusion-related activities (€8.06), and advanced wound care (€4.67).

### 3.4. Material Costs

More than 1000 distinct material items were identified from Phase 2. The mean material cost per home visit was €6.50.

### 3.5. Cost per Patient per Day

Table 4 reports the mean daily accounting cost per patient, before and after including the nursing activities performed during the home visit. Regarding the base-case scenario, the mean cost amounted to €27.78 per patient per day. Travel-related costs (nursing time spent travelling and vehicle costs) represented the largest cost component, followed by nursing time at the patient’s home and material costs. Under the extended scenario, the inclusion of the estimated economic value of three nursing activities per visit (€93.03) increased the estimated daily cost per patient to €120.81.

### 3.6. Cost per Nurse per Day

At the provider level, assuming a mean caseload of 6.84 patients per shift, the total daily accounting cost per nurse was €190.00. When the economic value of nursing activities was included, the estimated daily cost per nurse increased to €826.32 (Table 5).

### 3.7. Sensitivity Analyses

**Figure 1:**
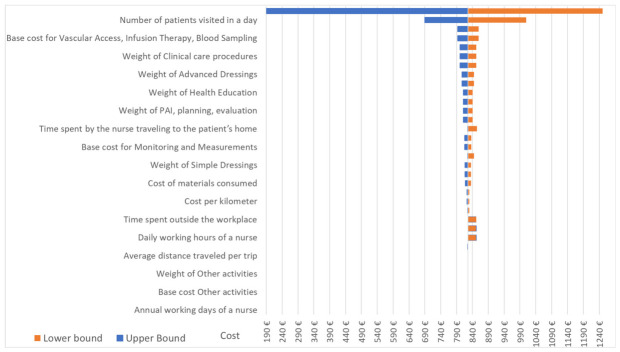
Deterministic sensitivity analysis of mean daily cost per nurse: tornado diagram showing the relative impact of key input parameters on cost estimates. Note. The tornado diagram summarizes the results of the deterministic sensitivity analysis, showing the relative impact of key input parameters on estimated daily costs. Wider bars indicate parameters with a greater influence on the estimated daily cost.

**Figure 2:**
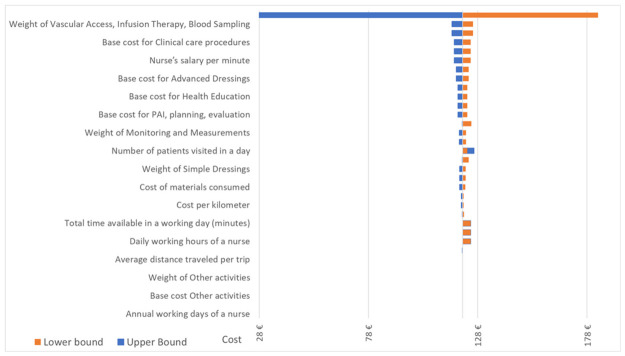
Deterministic sensitivity analysis of mean daily cost per patient: tornado diagram showing the relative impact of key input parameters on cost estimates. Note. The tornado diagram summarizes the results of the deterministic sensitivity analysis, showing the relative impact of key input parameters on estimated daily costs. Wider bars indicate parameters with a greater influence on cost variability.

Deterministic sensitivity analysis identified the number of patients visited per shift and the number of activities performed per visit as the parameters with the greatest influence on total daily costs. Variations in material costs and transportation-related expenses had a comparatively limited impact (Figure 1 and Figure 2).

Probabilistic sensitivity analysis confirmed the robustness of the model. The mean estimated daily cost per patient remained close to the base-case value, with a moderately right-skewed distribution and no evidence of extreme instability. The corresponding results for daily costs per nurse are reported in Table 6.

### 3.8. Contextual Comparison of Home Care Nursing Costs with Hospital-Based Care

This analysis is presented as an exploratory and illustrative exercise intended to contextualize cost estimates; therefore, it does not constitute a formal budget impact or cost-effectiveness analysis. The estimated unit cost of a home care nursing visit was €27.78 in the base-case scenario and €120.81 when the illustrative economic value of nursing activities was included. At the provider level, the corresponding daily cost per nurse was €190.00 and €826.32, respectively. To contextualize these estimates at the system level, national epidemiologic and cost data were applied. According to AGENAS, 1,546,443 patients received home care services in Italy in 2024 [30]. Evidence from a systematic review and meta-analysis indicates that the availability of home care services (and specifically the hospital-at-home intervention) is associated with a 26% reduction in the risk of readmission, with a relative risk (RR) of 0.74 in acutely ill patient populations [31]. To account for the heterogeneity of the long-term home-care population (in which the avoidable-hospitalization effect is plausibly smaller than in acute hospital-at-home cohorts), we present a sensitivity range with RR varying from 0.74 to 0.95. Under the conservative RR = 0.95 scenario, the implied relative increase in hospitalization risk is approximately 5.26%, yielding approximately 81,392 additional hospitalizations and corresponding additional expenditure of approximately €356,552,781.6 per year. The resulting system-level expenditure range is therefore approximately €356,552,781.6–2,380,230,731 per year (single-hospitalization assumption), with an upper bound under the two-hospitalization assumption of approximately €713,105,563.2–4,760,461,462 per year. In the absence of home care, this corresponds to National data reporting an average cost of €617 per inpatient hospital day and a mean length of stay of 7.1 days [32], resulting in an approximate cost of €4380.70 per hospitalization. Applying the increased risk scenario to the national home care population yields an illustrative estimate of €2,380,230,731, corresponding to €356,552,781.6 under more conservative assumptions (lower RR and only one hospitalization per patient per year). These are additional costs considering the cost of resource utilization in healthcare without home care (e.g., the patient has to frequently access emergency services, general/nurse practitioners, outpatient visits, drugs, regular hospital admissions and rehabilitation programmes).

These estimates were compared with the annual cost of providing home care nursing based on AIDOMUS-IT data. Assuming an average of 1.5 home visits per patient per week (78 visits per year) and a mean accounting cost of €27.78 per visit, the estimated annual cost per patient is €2166.84. Applied to the national population receiving home care, the total annual expenditure can be approximately €3.35 billion. If a cost of €120.81 of is considered, the estimated annual cost per patient is €9423.18. These annual estimates, compared with healthcare service utilization as reported above, represent a reduced cost for patient management.

## 4. Discussion

The main objective of this study was to estimate the accounting cost of home care nursing in Italy and to identify the main cost drivers influencing expenditure in home care. To the best of our knowledge, this is the first national micro-costing study of home care nursing conducted in Italy. These results represent an illustrative scenario rather than a formal budget impact analysis, and results are sensitive to assumptions regarding hospitalization rates, survey responses and the availability of data on home care services in Italy. Providing empirically grounded cost estimates may support healthcare planning and resource allocation decisions within the Italian National Health Service.

The analysis demonstrates a substantial discrepancy between accounting-based estimates and the more comprehensive valuation that includes nursing activities. The base-case scenario represents the minimal operational cost of home-care nursing activity (€27.78 per patient per day), while the extended scenario underscores the substantial economic value of nursing activities currently unaccounted for in reimbursement schemes (€120.81 per patient per day). At the organizational level, the inclusion of activities increases the estimated daily cost per nurse from €190.00 to €826.32, highlighting a structural underestimation of the economic contribution of home-care nursing within existing funding models. These findings mark the importance of developing an appropriate reimbursement framework for home-based nursing care that reflects both the direct operational costs and the clinical value of the activities delivered. Addressing the lack of an appropriate reimbursement framework is therefore essential to ensure the long-term viability and quality of home care delivered. The observation that a substantial proportion of visits include multiple concurrent activities, alongside a high volume of non-procedural tasks such as documentation, care planning, and patient education, further underscores the multidimensional nature of home care nursing and challenges simplified representations of home care as low-intensity care. It is essential to clarify that the base case and the extended scenario measure two conceptually distinct economic quantities and are therefore not directly additive. The base case (€27.78 per patient per day) is a bottom-up accounting cost capturing the resources actually consumed in delivering one home visit, valued at their respective input prices. The extended scenario (€120.81 per patient per day) is a value-attribution exercise that asks how much the same set of nursing activities would be reimbursed if paid through the existing outpatient tariff schedule; because outpatient tariffs are regulatory prices that bundle nursing labour, ancillary materials, equipment depreciation, and indirect overheads into a single price, they necessarily embed the cost of professional nursing time. The €120.81 figure should therefore be interpreted as the upper-bound regulatory value of the visit under the existing outpatient framework, not as the true production cost. The substantial gap between the two values reflects the absence of a dedicated reimbursement mechanism for home-based nursing care that would adequately remunerate its complexity, multidimensional nature, and contextual demands. The findings also indicate that activity intensity represents a major driver of costs in home care nursing. In the extended scenario, variations in the number and complexity of activities performed during a single visit may substantially influence overall cost estimates. Although the assumption of three activities per visit was empirically grounded in the observed data, home care visits ranged from lower-intensity encounters involving one or two activities to more complex visits including multiple concurrent interventions. These findings highlight the multidimensional nature of home care nursing and suggest that differences in care complexity should be considered when interpreting and transferring cost estimates across organizational contexts. These findings should be interpreted considering the broader international literature, which highlights both the growing relevance of home-based care and the limited availability of robust economic evaluations. Previous studies have shown that home care interventions are associated with improved patient adherence, satisfaction, and quality of life, and in some cases with reductions in healthcare utilization, although the economic evidence remains heterogeneous and strongly context-dependent. In this field, the present study contributes to filling an important gap by providing detailed, bottom-up cost estimates based on real-world data, an approach that remains relatively uncommon in the field. The most directly comparable international micro-costing studies on home-based nursing care to date are the Korean analyses by Ryu [20], which estimated the cost per home care nursing visit using administrative and activity-based data. After conversion to 2024 euros and adjustment for purchasing power parity, the Korean estimates correspond to approximately €30–45 per visit, broadly consistent with our €27.78 base-case figure. Direct comparability is however limited by several factors: (i) the Korean studies were conducted in a privately insured payment-by-tariff system that already incorporates a dedicated reimbursement scheme for home nursing visits (unlike Italian Public Healthcare Systems); (ii) the scope of activities included in the Korean cost definitions differs from the AIDOMUS-IT activity classification (notably with respect to travel time, which is incorporated differently in the two systems); (iii) price levels and labour-cost structures differ substantially. With these considerations, the AIDOMUS-IT base case is in broad alignment with the Korean precedent, providing the first empirical evidence that the order of magnitude of home nursing visit costs in Italy is comparable to that observed in the closest international precedent. From a methodological perspective, the costing strategy adopted in this study is consistent with ingredient-based approaches used in other healthcare settings, in which total costs are derived from the aggregation of labour and material inputs required to deliver services. At the same time, international evidence emphasizes that the economics of home care are strongly influenced by organizational and workforce-related factors. More in detail, labour costs typically represent the largest component of total expenditure, and workforce availability, stability, and skill mix are key determinants of service sustainability. These considerations are consistent with the present findings, which highlight the role of variables such as caseload, visit duration, and care complexity in shaping cost estimates. A key aspect of this analysis concerns the use of outpatient service definitions and tariffs as a proxy for assessing nursing activities in the extended scenario. This approach has represented a pragmatic solution due to the absence of a dedicated reimbursement system for HCN in Italy. Such adoption introduces a few conceptual limitations. First, outpatient tariffs are designed to reflect discrete and procedure-based activities, whereas HCN involves integrated and context-dependent care dynamics delivered within a single visit. As a result, the use of outpatient tariffs may not fully capture the complexity, continuity, and organizational dimensions of home-based nursing care. These limitations are not only methodological but also have relevant system-level implications. The absence of dedicated reimbursement mechanisms for nursing activities risks systematically undervaluing the contribution of HCN within territorial health systems and may hinder the strategic development of community-based services. Furthermore, the strong influence of organizational variables such as caseload, visit duration, and care complexity suggests that workforce planning strategies may be as important as wage levels in determining the sustainability and efficiency of home care services. The economic analysis suggests that home care nursing services may represent a potentially efficient model of care. Given the ageing Italian population and the increasing prevalence of chronic diseases [33], investment in home care represents a sustainable and strategic allocation of healthcare resources, as reported by international literature [14,34]. Previous studies suggest that home care may contribute to reducing unnecessary hospital readmissions, mitigating complications related to hospital stays, enhancing patient satisfaction, and supporting care continuity [17], although these outcomes were not directly evaluated in the present study. The exploratory comparison suggests that the potential economic implications of home care may be substantial; however, these estimates are based on external data and simplifying assumptions and should be interpreted with caution [35]. Future research should integrate health outcomes into economic evaluations to enable full cost-effectiveness analyses and better inform value-based decision making. Multicenter studies capturing broader regional variability are needed to assess the transferability of cost estimates across different organizational contexts. In addition, future prospective studies should explore the relationship between home care intensity, patient complexity and healthcare utilization, including hospital admissions and long-term care as patients’ outcomes. Further refinement of activity-based costing and standardized data collection frameworks would also strengthen comparability across studies and support evidence-informed reimbursement policies. Strengthening home-care capacity (through workforce expansion, digital support tools, improved logistics, and structured clinical pathways) should therefore be considered a national priority for cost containment, quality improvement, and long-term sustainability of the SSN. Moreover, the cost of home care compared to the related cost of hospital care if home care fails to be provided highlights a compelling economic argument: home-based nursing care is clinically valuable and patient-centred, and may have important economic implications for the SSN; however, the estimates provided in this study should be interpreted as illustrative and not as evidence of cost-effectiveness or budget impact.

### Limitations

Some limitations should be considered. First, in the absence of an official fee schedule for home-based nursing services, unit costs were derived from the specialist outpatient medical tariff schedule. Although this approach provided a standardized reference, it may not accurately reflect the true economic value of nursing activities, leading to cost inaccuracies or overestimation. Second, material costs are characterized by substantial variability across local contexts, procurement systems, and organizational practices. The estimates used in this study should therefore be interpreted as indicative rather than definitive, and the transferability of these values to other settings may be limited. Third, the analysis relied on assumptions due to incomplete data availability. These assumptions were informed by expert consultation and available evidence, which may reduce but cannot eliminate the risk of bias. In particular, the economic model did not incorporate health outcomes and was therefore limited to a cost analysis rather than a full cost-effectiveness evaluation. Fourth, the estimated number of patients receiving home care may be underestimated, as available data referred primarily to individuals aged over 65 years. Fifth, the nursing sample may not perfectly represent the broader population of professionals involved in home care delivery, potentially affecting the generalizability of workload and cost estimates. Sixth, the time-and-motion observations used to estimate visit duration were conducted in a limited number of LHAs and may not fully capture the variability of organizational models and service delivery patterns across the entire Italian healthcare system. A further limitation concerns the allocation of material costs. Material consumption was collected and costed at the visit level, while each visit could include multiple nursing procedures. As a result, it was not always possible to attribute specific materials to individual procedures. This does not affect the estimation of the average material cost per visit but limits the interpretation of procedure-specific costs, including materials. These estimates should therefore be interpreted as visit-level averages rather than direct procedure-level material costs. On the other hand, extensive sensitivity analyses were conducted to test the robustness of the model assumptions. A wide variability range (±30%) was applied to all key parameters, increasing confidence in the stability of the results despite uncertainty in the underlying inputs. Another limitation is that this study adopted the perspective of the Italian National Health Service and therefore did not include indirect costs, such as caregiver burden, informal care time, or productivity losses. While this approach is appropriate for informing healthcare planning and resource allocation within the public system, it limits the interpretation of results from a broader societal perspective. Finally, the analysis does not account for specific organizational aspects such as unsuccessful interventions, extended follow-up pathways, or alternative remuneration models, which may influence cost structures in different settings.

## 5. Conclusions

This national micro-costing study provides a first picture of empirically grounded estimates regarding home care nursing costs in Italy, and it identifies organizational factors as the primary drivers of expenditure. These estimates are derived from visit-level costing and translated into daily cost indicators to support policy and organizational decision-making. The findings highlight a substantial gap between operational costs and the broader economic value of nursing activities, reflecting the absence of a dedicated reimbursement framework for home-based care. Despite operating at relatively low direct costs, home care nursing delivers complex, multidimensional services that are essential for continuity of care and system sustainability. While this analysis suggests potential system-level savings, these estimates should be interpreted cautiously, considering the underlying assumptions and methodological limitations.

## Figures and Tables

**Table 1:** Characteristics of the nurses participating in Phase 1 (n = 3949).

Sample Characteristics	Frequency (%)	Mean (SD)
Age	--	46.02 (10.23)
Sex		
Male	784 (19.85)	--
Female	3088 (78.20)	--
Prefer not to say	77 (1.95)	--
Highest educational qualification held (n = 3944)		
Regional diploma	1674 (42.44)	--
Diploma	284 (7.20)	--
Bachelor’s degree	1813 (45.97)	--
Master’s degree	173 (4.39)	--
Postgraduate course in home nursing or family nurse care		
Yes	1023 (25.91)	--
No	2926 (74.09)	--
Home visits in the last shift		6.84 (3.14)
Years of experience as a home nurse practitioner (n = 3887)	--	8.01 (8.26)

Legend. SD, standard deviation.

**Table 2:** Characteristics of home visits and the type of nursing activities performed in Phase 2.

Visits Characteristics	Median (25th–75th Percentile)
Time to reach the patient’s home (minutes)	10 (5–15)
Distance travelled to reach the patient’s home (km)	3.5 (2–7)
Number of nursing activities performed per visit	3 (2–4.5)
	n (%)
Distribution of visits by number of activities	
Number of visits with 3 or more nursing activities performed	314 (59.58)
Number of visits with 2 or fewer nursing activities performed	213 (40.42)
Types of nursing activities performed during home visits,	
Nursing assessment *	269 (51.04)
Procurement of medical supplies/medications	75 (14.23)
Dressings and/or bandages	110 (20.87)
Telephone consultation for health support **	22 (4.17)
Documentation (individualized care plan, nursing record, assessment scales, back-office forms)	467 (88.62)
Counselling (individual or group)	26 (4.93)
Health education ***	185 (35.1)
Central venous catheter management	42 (7.97)
Urinary catheter management	32 (6.08)
PEG/PEJ or enterostomy management	20 (3.8)
Wound dressing (pressure, neoplastic, vascular, diabetic foot, surgical, or traumatic lesions)	160 (30.36)
Blood sampling	92 (17.46)
Measurement of vital signs	35 (6.64)
Administration of medications and solutions	72 (13.67)
Other ****	100 (18.98)

Legend. PEG, percutaneous endoscopic gastrostomy; PEJ, percutaneous endoscopic jejunostomy; SD, standard deviation. Notes. * Intake assessment, evaluation of self-care, nursing consultation, etc., ** Communication, device management, interventions, etc., *** Patient/caregiver education regarding the performance of healthcare activities, medication administration, and use of medical devices/equipment. **** Fundamental nursing care, subcutaneous needle placement, patient mobilization, urine sample collection, burn dressing, drain removal, microbiological swab, bronchial aspiration, electrocardiogram, enteral nutrition administration, enema, continuous glucose monitoring adjustment, peripheral venous catheter insertion.

**Table 3:** Distribution and weighted average cost of nursing activity categories.

Category	Unit Cost (€)	Frequency (%)	Weighted Cost (€)
Vascular access, infusion therapy, and blood sampling	38.06	21.17	8.06
Health education	15	23.97	3.60
Advanced wound care	39.13	11.93	4.67
Simple wound care	29.72	7.89	2.34
Monitoring and measurements	40.95	6.2	2.54
ICP, planning, evaluation	33.81	10.45	3.53
Clinical care activities	34.1	18.39	6.27
Total	—	100	31.01

Note. Weighted costs were calculated by combining the relative frequency of nursing activity categories (derived from Phase 1) with their corresponding unit costs. Percentages refer to the distribution of nursing activities, not to the proportion of home care visits. Legend. ICP, individual care plan.

**Table 4:** Mean daily cost per patient under base-case and extended scenario.

Cost Item	Base-Case Scenario (€)	Extended Scenario (€)
Nursing time at patient’s home	6.95	6.95
Travel to patient’s home	10.25	10.25
Return travel to workplace	1.5	1.5
Back-office activities	0.04	0.04
Medical materials	6.5	6.5
Transport (vehicle)	2.54	2.54
Subtotal (accounting costs)	27.78	27.78
Nursing activities (3 per visit)	–	93.03
Total per patient per day	27.78	120.81

**Table 5:** Mean daily cost per nurse under base-case and extended scenario.

Cost Item	Base-Case Scenario (€)	Extended Scenario (€)
Nursing time at patient’s home	47.53	47.53
Travel to patients’ homes	70.14	70.14
Return travel to workplace	10.25	10.25
Back-office activities	0.25	0.25
Medical materials	44.47	44.47
Transport (vehicle)	17.36	17.36
Subtotal (accounting costs)	190	190
Nursing activities (3 per patient)	–	636.32
Total per nurse per day	190	826.32

Note. The extended scenario includes an illustrative valuation of nursing activities based on the maximum outpatient tariffs and does not represent an official reimbursement scheme.

**Table 6:** Probabilistic sensitivity analysis results for daily home care nursing costs.

Outcome	Mean (€)	Standard Deviation (€)	Minimum (€)	2.5th Percentile (€)	97.5th Percentile (€)	Maximum (€)
Cost per nurse per day	870.85	350.16	178.73	386.37	1729.77	3375.37
Cost per patient per day	131.56	36.4	37.96	73.6	217.12	450.37

Note. Results are derived from a Monte Carlo simulation with 10,000 iterations. Values represent the distribution of estimated costs under simultaneous uncertainty of all model parameters. Costs are expressed in euros (€).

## Data Availability

The data that support the findings of this study are not publicly available due to privacy and ethical restrictions but are available from the corresponding author upon reasonable request, subject to approval by the relevant ethics committee.

## Supplementary Materials

The following supporting information can be downloaded at: https://www.mdpi.com/article/10.3390/nursrep16060180/s1, CHEERS 2022 Checklist [36].

## Public Involvement Statement

No public involvement in any aspect of this research.

## Guidelines and Standards Statement

This study was reported in accordance with the Strengthening the Reporting of Observational Studies in Epidemiology (STROBE) guidelines. In addition, elements of the Consolidated Health Economic Evaluation Reporting Standards (CHEERS 2022) were considered to ensure transparent reporting of the economic analysis. A complete list of reporting guidelines can be accessed via the equator network: https://www.equator-network.org/.

## Abbreviations

The following abbreviations are used in this manuscript:

HCN	Home care nursing
LHA	Local Health Authorities
SSN	Servizio Sanitario Nazionale, Public Italian Healthcare Service
