# Supplementary material for: How Much Does a Home Care Nursing Visit Cost? A National Micro-Costing Study from the AIDOMUS-IT Project
Source: Nurs Rep. 2026 May 26;16(6):180. doi: 10.3390/nursrep16060180 (PMC13304600; doi:10.3390/nursrep16060180)
Supplement: Supplementary file 1 [file nursrep-16-00180-s001.zip › nursrep-4252706-supplementary.pdf]

Supplementary Material

CHEERS 2022 Checklist

This document presents the completed CHEERS 2022 checklist for the AIDOMUS-IT micro-costing study. The CHEERS 2022 statement comprises 28 items organised into seven sections (Title, Abstract, Introduction, Methods, Results, Discussion, Other relevant information). In line with the CHEERS 2022 guidance, items that do not apply to a cost analysis (i.e., items 11–13 for outcome measurement and valuation, and item 16 for decision-analytic modelling) are reported as 'Not applicable' with an explicit rationale. Item 18 (heterogeneity), item 19 (distributional effects), and item 21 (patient/public engagement) are reported according to their relevance to the present study. Section references refer to the revised manuscript (version 2).

Section / Topic	Item No	Guidance for reporting	Reported in section
**TITLE**			
**Title**	**1**	Identify the study as an economic evaluation and specify the interventions being compared.	Title (page 1): "How much does a home care nursing visit cost? A national micro-costing study from the AIDOMUS-IT project". The title identifies the study as a micro-costing analysis. NOTE: This is a cost analysis (production-cost estimation) rather than a comparative economic evaluation with two alternative interventions; the AIDOMUS-IT study estimates the resource consumption of home care nursing as delivered in Italy and explores an illustrative comparison with hospital-based care in the Discussion. The title has been revised to sentence case in response to reviewer feedback.
**ABSTRACT**			
**Abstract**	**2**	Provide a structured summary that highlights context, key methods, results, and alternative analyses.	Abstract (page 2). The structured abstract reports: Background/Objectives (demographic and epidemiological context, gap in Italian micro-costing evidence, study aim), Methods (multicentre cross-sectional design; two-phase data collection, n = 3,949 nurses and n = 527 time-and-motion observations; ingredient-based bottom-up costing; deterministic and probabilistic sensitivity analyses), Results (base-case €27.78 per patient per day; extended scenario €120.81; cost drivers identified), and Conclusions (policy implications and reimbursement framework).
**INTRODUCTION**			
**Background and objectives**	**3**	Give the context for the study, the study question, and its practical relevance for decision making in policy or practice.	Section 1 "Introduction" (pages 2–3). The context is articulated across four paragraphs: (i) ageing population and chronic-disease burden in Italy; (ii) the 2022 national reform on territorial care and the role of home care

			nursing in community-based service delivery; (iii) the international evidence base on cost-effectiveness of home care, mostly modelling-based and context-dependent; (iv) the absence in Italy of a dedicated tariff for home-based nursing activities, justifying the need for empirical micro-costing evidence. Section 1.1 ("Aims of the study") provides an explicit statement of the two primary objectives (estimate accounting cost; identify cost drivers) and one secondary objective (illustrative comparison with hospital-based care).
**METHODS**			
**Health economic analysis plan**	**4**	Indicate whether a health economic analysis plan was developed and where available.	A formal pre-registered health economic analysis plan was NOT developed as a standalone document. The economic analysis is a secondary analysis of the AIDOMUS-IT project, whose overall protocol is published as Bagnasco et al. 2023 (BMJ Open, reference [21] in the manuscript). The costing methodology is described in detail in Sections 2.4–2.11 of the revised manuscript and follows the ingredient-based bottom-up micro-costing approach standardly applied in healthcare research (Tan et al. 2009, reference [18]). NOTE FOR FUTURE STUDIES: development of a stand-alone health economic analysis plan, registered before data analysis, is recommended.
**Study population**	**5**	Describe characteristics of the study population (such as age range, demographics, socioeconomic, or clinical characteristics).	Section 2.2 "Population and Setting" (pages 3–4) and Section 3.1 "Sample characteristics" with Table 1 (page 6). Phase 1: 3,949 registered nurses (mean age 46.0 ± 10.2 years; 78.2% female; 46.0% with bachelor's degree; 25.9% with postgraduate training in home/family nursing; 8.0 ± 8.3 years of experience). Phase 2: a sub-sample of 83 nurses across 3 LHAs in Liguria and Tuscany. Patient characteristics: although the broader AIDOMUS-IT study included patient-level data, the present economic analysis used nurse-reported and time-and-motion data only; patient-level stratification by age, complexity, or dependency was not performed (this is explicitly stated in Section 2.5 and acknowledged as a limitation).
**Setting and location**	**6**	Provide relevant contextual information that may influence findings.	Section 2.1 "Study design" and Section 2.2 "Population and Setting" (pages 3–4). Setting: Italian public home care nursing services delivered by Local Health Authorities (LHAs) under the National Health Service (SSN). Phase 1 covered 70 LHAs across 18 of the 21 Italian regions (April–October 2023). Phase 2 covered 3 LHAs in Liguria and Tuscany (March 2024), selected purposively to capture variability in organisational models. The Italian regulatory context, characterised by the absence of a national tariff for

			home-based nursing activities and substantial regional heterogeneity in funding and organisation, is explicitly described in Section 1 and re-discussed in Section 4 (Discussion).
**Comparators**	**7**	Describe the interventions or strategies being compared and why chosen.	*NOT APPLICABLE in the strict comparative sense, as this is a cost analysis estimating the production cost of an existing service (home care nursing) rather than comparing two alternative interventions. The illustrative analysis in Section 3.8 compares the estimated annual cost of providing home care nursing to the entire Italian home-care population (1,546,443 patients) with an exploratory estimate of the additional hospitalisation expenditure that would occur in the counterfactual scenario "no home-care availability" (using a meta-analytic RR = 0.74 from hospital-at-home interventions). This comparison is explicitly framed as illustrative and external-data-based, not as a cost-effectiveness analysis.*
**Perspective**	**8**	State the perspective(s) adopted by the study and why chosen.	Section 2.5 "Costing approach" (page 4). The analysis adopts the perspective of the Italian National Health Service (SSN) and includes direct healthcare costs only (personnel time, materials, transportation, back-office activities). Indirect costs (caregiver burden, informal-care time, productivity losses, out-of-pocket costs) are NOT included. This perspective was chosen because: (i) home care nursing in Italy is fully publicly funded; (ii) the primary policy audience of the study is the SSN, including national and regional planners; and (iii) the absence of a dedicated tariff for nursing activities is a system-level (SSN) issue. The exclusion of the societal perspective is explicitly acknowledged as a limitation in Section 4.1 (Limitations).
**Time horizon**	**9**	State the time horizon for the study and why appropriate.	Section 2.5 (revised) "Costing approach": the analysis adopts a one-year time horizon. This horizon was selected because: (i) home care nursing visits are recurrent services with annual planning and budgeting cycles aligned with the SSN fiscal year; (ii) the variables of interest (caseload, travel patterns, materials consumption) are aggregated as annual flows; (iii) the study does not include longer-term outcomes that would justify multi-year extrapolation. No discounting is required given the short horizon (see item 10).
**Discount rate**	**10**	Report the discount rate(s) and reason chosen.	*NOT APPLICABLE — given the one-year time horizon of the analysis (see item 9), discounting of costs is not required. All costs are valued at their 2024 nominal value and no inter-temporal adjustment is applied. This is now explicitly stated in Section 2.5 of the revised manuscript.*

**Selection of outcomes**	**11**	Describe what outcomes were used as the measure(s) of benefit(s) and harm(s).	*NOT APPLICABLE per CHEERS 2022 guidance for cost analyses. The present study is a cost analysis and does not measure health benefits or harms (such as QALYs, DALYs, life-years, or clinical outcomes) since no comparator alternative is evaluated. The output of the analysis is the per-visit, per-patient-per-day, and per-nurse-per-day cost of home care nursing. The Discussion explicitly emphasises that future research integrating health outcomes is needed to perform full cost-effectiveness analyses.*
**Measurement of outcomes**	**12**	Describe how outcomes used to capture benefit(s) and harm(s) were measured.	*NOT APPLICABLE per CHEERS 2022 guidance for cost analyses (see item 11).*
**Valuation of outcomes**	**13**	Describe the population and methods used to measure and value outcomes.	*NOT APPLICABLE per CHEERS 2022 guidance for cost analyses (see item 11).*
**Measurement and valuation of resources and costs**	**14**	Describe how costs were valued.	Sections 2.5–2.11 (pages 4–5). Five cost components were measured and valued: (i) Personnel time (Section 2.6) — nursing time was valued using the per-minute labour cost derived from the 2019–2021 National Collective Labour Agreement for the public healthcare sector (CCNL Sanità), salary profile D, band D4, assuming 260 working days per year and 7.2 hours per day. The D4 assumption is now explicitly identified as a simplification and tested in sensitivity analysis (D3, D5, Ds profiles). (ii) Back-office activities (Section 2.7) , documentation, care planning, coordination valued using the same per-minute labour cost. (iii) Material costs (Section 2.8), more than 1,000 distinct items grouped into 6 categories; unit prices from the National Pharmaceutical Formulary (AIFA) and regional public procurement catalogues (Liguria, Tuscany); costs measured at the visit level. (iv) Transportation (Section 2.9), vehicle costs valued using 2024 ACI per-kilometre rates; travel time valued using the per-minute labour cost; Phase 1 cumulative per-shift travel time (34.7 min) used for daily cost build-up, with Phase 2 per-segment travel time (median 10 min) used descriptively. (v) Activity-tariff valuation in the extended scenario (Section 2.10), using maximum (primary scenario) and minimum and median (sensitivity) outpatient tariffs from the Italian Ministry of Health 2023 fee schedule. The conceptual distinction between bottom-up accounting cost (base case) and tariff-based regulatory value (extended scenario) is now made explicit (see also items 17 and 26).
**Currency, price date, and conversion**	**15**	Report the dates of the estimated resource quantities and unit costs, plus the currency and year of conversion.	Section 2.5 (revised) "Costing approach". Currency: Euro (€). Price date: 2024, all costs are expressed in 2024 euros. Resource quantities: Phase 1 data collected April–October 2023; Phase 2 time-and-motion data collected March 2024. Wage values from the 2019–2021 CCNL were inflation-

			adjusted to 2024 using the official ISTAT revaluation index (https://rivaluta.istat.it/). Material costs based on 2024 AIFA and regional procurement catalogues. Transportation costs from the 2024 ACI cost-per-kilometre tables. No currency conversion was applied (single-country study). In the Discussion, the Korean reference estimates by Ryu (2009) and Ryu et al. (2006) are converted to 2024 euros using purchasing power parity (PPP) for comparability.
**Rationale and description of model**	**16**	If modelling is used, describe in detail and why used. Report if the model is publicly available and where it can be accessed.	*NOT APPLICABLE per CHEERS 2022 guidance for non-modelling studies. The present study is empirical and uses primary data collection (Phase 1 survey, Phase 2 time-and-motion observations) plus deterministic and probabilistic sensitivity analysis around the directly measured central values. No decision-analytic model (e.g., Markov, decision tree, microsimulation) is constructed. The Monte Carlo simulation in Section 2.11 is a probabilistic uncertainty analysis around the empirical micro-costing parameters, not a structural decision model.*
**Analytics and assumptions**	**17**	Describe any methods for analysing or statistically transforming data, any extrapolation methods, and approaches for validating any model used.	Section 2.10 "Costing scenarios" and Section 2.11 "Sensitivity analysis" (page 5). Data analysis: (i) Phase 1 data analysed descriptively to characterise the workforce, workload, and activity distribution; the relative frequency of each of the seven activity categories was computed as the number of activities in the category divided by the total number of activities (10,972), and used as weights in computing the weighted-average cost of nursing activities. (ii) Phase 2 data analysed descriptively to characterise visits (median, IQR for skewed variables; mean ± SD for symmetric variables); material costs aggregated at the visit level. (iii) Daily cost build-up: per-visit cost × visits-per-shift = per-nurse cost; per-visit cost ÷ patients-per-nurse = per-patient cost. The two costing scenarios (base case = bottom-up accounting cost; extended scenario = tariff-based imputed regulatory value) are described in Section 2.10, to address the potential double-counting concern raised by the reviewer. No extrapolation across populations or time horizons was performed. No external model was validated.
**Characterising heterogeneity**	**18**	Describe any methods used for estimating how the results of the study vary for subgroups.	PARTIALLY REPORTED. The present analysis does not stratify cost estimates by patient subgroups (age, clinical complexity, diagnosis, dependency), since the analysis relied on nurse-reported and time-and-motion data rather than on patient-level cost data; this limitation is explicitly acknowledged in Section 2.5 and Section 4.1 (Limitations). Heterogeneity is however explored in terms of: (i) activity intensity, the deterministic

			sensitivity analysis varies the number of activities per visit (Section 3.7 and Figures 1–2); (ii) salary band, a sensitivity check across CCNL bands D3, D5, Ds was added (see item 14); (iii) tariff choice, a parallel sensitivity scenario using minimum and median outpatient tariffs was added (Table 7); (iv) RR effect size, in the illustrative hospital comparison, a range RR = 0.74 to RR = 0.95 is reported.
**Characterising distributional effects**	**19**	Describe how impacts are distributed across different individuals or adjustments made to reflect priority populations.	NOT REPORTED in the present study. Equity and distributional effects across patient subgroups, regions, or socioeconomic strata are not formally analysed, as the focus of the present analysis is the average production cost of a home nursing visit at the national level. The Discussion acknowledges that substantial regional heterogeneity exists in the organisation and funding of home care services across Italian regions, and that this heterogeneity may affect both the actual cost and the patient population served. Formal distributional analysis is recommended for future work that combines AIDOMUS-IT cost data with patient-level outcome and equity data.
**Characterising uncertainty**	**20**	Describe methods to characterise any sources of uncertainty in the analysis.	Section 2.11 "Sensitivity analysis" (page 5) and Section 3.7 "Sensitivity analyses" (page 7), with Figures 1–2 (tornado diagrams) and Table 6 (PSA results). (i) Deterministic sensitivity analysis: each input parameter (number of patients per shift, number of activities per visit, nursing labour cost, material cost, travel time and distance) varied individually by ±30% (when empirical variability was unavailable); results presented as tornado diagrams. (ii) Probabilistic sensitivity analysis (PSA): Monte Carlo simulation with 10,000 iterations; gamma distributions for cost variables (parameterised to have mean equal to the base case and SD equal to 30% of the base case, yielding shape $\alpha \approx 11.11$ and rate $\beta = \alpha/\text{mean}$); beta distributions for proportion parameters (method-of-moments matching). Results reported as mean, SD, 2.5th and 97.5th percentiles, minimum and maximum. Supplementary Table S1 explicitly lists each model parameter with its base-case value, distribution family, distribution parameters, and rationale, addressing the reviewer's reproducibility concern. Sensitivity analyses on the salary band (D3, D4, D5, Ds), the tariff choice (minimum, median, maximum), and the RR effect size in the hospital comparison (0.74–0.95).
**Approach to engagement with**	**21**	Describe any approaches to engage patients or service recipients, the general public, communities, or stakeholders (such as clinicians or payers) in the design of the study.	The broader AIDOMUS-IT project involved the Italian national professional federation of nurses (FNOPI) through CERSI-FNOPI as scientific partner, and engaged Nursing Directors of the 70 participating LHAs as local facilitators

**patients and others affected by the study**			(Section 2.3). Direct patient and public involvement (PPIE) was NOT formally implemented in the design of the present economic analysis; the Public Involvement Statement of the manuscript explicitly states: "No public involvement in any aspect of this research." Engagement of patients and informal caregivers occurred only as respondents to broader AIDOMUS-IT survey instruments (Bagnasco et al. 2024, reference [5]) and did not contribute to the design of the economic analysis. This is acknowledged as a limitation.
**RESULTS**			
**Study parameters**	**22**	Report all analytic inputs (such as values, ranges, references) including uncertainty or distributional assumptions.	Reported across Section 3 ("Results") and Section 2 ("Methods"). Specifically: (i) Workforce parameters, Table 1 (Phase 1 nurse characteristics, including mean caseload of 6.84 patients per shift). (ii) Visit parameters, Table 2 (visit characteristics including median travel time, distance, and number of activities; activity-type frequencies). (iii) Activity unit costs and weighted average cost, Table 3 (unit cost, frequency, weighted cost for the 7 categories). (iv) Material costs, Section 3.4, reporting per-category mean, SD, IQR, and number of visits in which each category occurred. (v) Cost build-up per patient per day, Table 4 (base case and extended scenario). (vi) Cost build-up per nurse per day, Table 5 (base case and extended scenario). (vii) PSA inputs and distributions. (viii) PSA outputs, Table 6 (mean, SD, percentiles, min, max). (ix) Sensitivity scenarios Table 7 (min/median tariff scenarios) and supplementary salary-band sensitivity. References to underlying data sources (CCNL, AIFA, ACI, Ministero della Salute tariff schedule) are cited explicitly.
**Summary of main results**	**23**	Report the mean values for the main categories of costs and outcomes of interest and summarise them in the most appropriate overall measure.	Sections 3.3–3.6 (pages 6–7). Mean values reported: (i) Mean activity cost per visit: €31.01 (weighted average across the 7 categories, Table 3). (ii) Mean material cost per visit: €6.50 (Section 3.4). (iii) Mean daily cost per patient: €27.78 (base case) and €120.81 (extended scenario). (iv) Mean daily cost per nurse: €190.00 (base case) and €826.32 (extended scenario), corrected for double-counting framing in Table 5. These figures are the main results of the analysis and are summarised in the Abstract and re-stated in the Conclusions. The illustrative system-level annual estimates (€3.35 billion under the base case; €14.572 billion under the extended scenario) are reported in the Discussion as exploratory contextual estimates.

**Effect of uncertainty**	**24**	Describe how uncertainty about analytic judgments, inputs, or projections affect findings. Report the effect of choice of discount rate and time horizon, if applicable.	Section 3.7 "Sensitivity analyses" (page 7) and revised Discussion. Effects of uncertainty: (i) Deterministic: tornado diagrams (Figures 1–2) show that number of patients per shift and number of activities per visit are the dominant cost drivers; material and transport costs have comparatively limited impact. (ii) Probabilistic (Table 6): mean cost per patient per day = €131.56 (SD €36.40; 95% UI €73.60–€217.12); mean cost per nurse per day = €870.85 (SD €350.16; 95% UI €386.37–€1,729.77). These intervals are reasonably tight around the base-case point estimates, supporting robustness. (iii) Salary band sensitivity: per-minute labour cost varies by approximately ±10% across CCNL bands D3, D4, D5, Ds, with negligible qualitative impact. (iv) Tariff choice sensitivity (Table 7): minimum/median outpatient tariffs yield substantially lower extended-scenario values, reframing the "headline gap" as a range. (v) RR effect size sensitivity: RR = 0.74 to RR = 0.95 yields an illustrative system-level expenditure range of approximately €0.36–€2.38 billion per year (single-hospitalisation assumption). Discount rate and time horizon do not apply (see items 9–10).
**Effect of engagement with patients and others affected by the study**	**25**	Report on any difference patient/service recipient, general public, community, or stakeholder involvement made to the approach or findings of the study.	NOT REPORTED. As stated in item 21, direct PPIE was not implemented for the present economic analysis. Engagement of the FNOPI scientific committee and Nursing Directors of the participating LHAs influenced the operational design of the data collection (e.g., feasibility of the time-and-motion forms, recruitment strategy) but did not formally affect the analytical choices of the economic analysis. This is explicitly acknowledged.
**DISCUSSION**			
**Study findings, limitations, generalisability, and current knowledge**	**26**	Report key findings, limitations, ethical or equity considerations not captured, and how these could affect patients, policy, or practice.	Section 4 "Discussion" (pages 7–9) and Section 4.1 "Limitations" (page 9), structured into three thematic domains. Key findings: substantial gap between operational base-case cost (€27.78 per patient per day) and tariff-based regulatory value (€120.81 per patient per day), reflecting absence of dedicated reimbursement framework; organisational factors (caseload, activity intensity) are dominant cost drivers; explicit comparison with Korean precedent studies. Limitations (revised structure): (a) Data-source limitations, Phase 1 self-reported data; Phase 2 limited to 3 LHAs (convenience sample); travel-time construct differences between phases; reliance on AIFA pricing with regional variability. (b) Methodological limitations, tariff-based proxy for the extended scenario; D4 wage band as proxy for the actual seniority distribution; visit-level material costing rather than procedure-level; one-year horizon without discounting; absence of integration of health outcomes (precluding cost-effectiveness analysis). (c)

			Generalisability limitations, convenience sampling at the LHA level for Phase 2; potential under-coverage of acute sub-populations in the illustrative comparison; SSN-only perspective; exclusion of indirect/informal-care costs. Ethical/equity considerations: regional heterogeneity in home-care funding and organisation across Italian regions may translate into inequitable access, which the cost estimates do not directly capture; formal distributional analysis is recommended for future work (see item 19). Policy and practice implications: the substantial gap between operational and regulatory cost supports the case for a dedicated national reimbursement framework for home-based nursing care; the dominance of organisational factors over unit prices supports workforce planning and operational optimisation as primary cost-containment levers.
**OTHER RELEVANT INFORMATION**			
**Source of funding**	**27**	Describe how the study was funded and any role of the funder in the identification, design, conduct, and reporting of the analysis.	Funding statement on page 9 of the manuscript: "This research received no external funding." The broader AIDOMUS-IT project was supported scientifically by CERSI-FNOPI (Centro di Eccellenza per la Ricerca e lo Sviluppo dell'Infermieristica, Federazione Nazionale degli Ordini delle Professioni Infermieristiche), as documented in the published study protocol (Bagnasco et al. 2023, reference [21]). No external commercial funder had any role in the design, conduct, analysis, interpretation, or reporting of the present economic analysis.
**Conflicts of interest**	**28**	Report authors' conflicts of interest according to journal or International Committee of Medical Journal Editors requirements.	Conflicts of Interest statement on page 9: "The funders had no role in the design of the study; in the collection, analyses, or interpretation of data; in the writing of the manuscript; or in the decision to publish the results." All authors declare no competing interests, in accordance with ICMJE requirements.

Legend and notes

- Yellow-shaded cells (NOT REPORTED / PARTIALLY REPORTED): items for which the requested information is not fully provided in the manuscript. For item 18 (heterogeneity), heterogeneity is explored only across the dimensions of activity intensity, salary band, and tariff choice, not across patient subgroups. For items 19 (distributional effects), 21 (PPIE), and 25 (effect of engagement), the present study does not formally implement these elements, which are explicitly acknowledged in the revised Discussion and Limitations.
- Grey-shaded cells (NOT APPLICABLE): items that do not apply to a cost analysis per CHEERS 2022 explicit guidance (items 11, 12, 13 for outcome measurement and valuation, and item 16 for decision-analytic modelling). Item 10 (discount rate) is also marked NOT APPLICABLE given the one-year time horizon. Item 7 (comparators) is partly NOT

APPLICABLE because the present analysis is not a comparative economic evaluation, but the illustrative system-level comparison with hospital-based care in the Discussion is described.

- White cells: items that are fully reported in the revised manuscript, with the specific Section and page reference indicated.
- In line with CHEERS 2022 guidance, the checklist is intended to capture quality of reporting, not quality of methods, and is not used as a scoring tool.

Source: Husereau D, Drummond M, Augustovski F, et al. Consolidated Health Economic Evaluation Reporting Standards 2022 (CHEERS 2022) Statement: updated reporting guidance for health economic evaluations. BMJ 2022;376:e067975. doi:10.1136/bmj-2021-067975. The CHEERS 2022 checklist is licensed under CC BY 4.0.
